# Six-Year Retrospective Analysis of Epidemiology, Risk Factors, and Antifungal Susceptibilities of Candidiasis from a Tertiary Care Hospital in South China

**DOI:** 10.1128/spectrum.00708-23

**Published:** 2023-06-13

**Authors:** Hazrat Bilal, Dongxing Zhang, Muhammad Shafiq, Muhammad Nadeem Khan, Canhua chen, Sabir Khan, Qian Wang, Lin Cai, Rehmat Islam, Haibin Hu, Yuebin Zeng

**Affiliations:** a Department of Dermatology, Second Affiliated Hospital of Shantou University Medical College, Shantou, China; b Department of Dermatology, Meizhou Dongshan Hospital, Meizhou, Guangdong Province, China; c Department of Dermatology, Meizhou People’s Hospital, Meizhou, Guangdong Province, China; d Department of Cell Biology and Genetics, Shantou University Medical College, Shantou, China; e Faculty of Biological Sciences, Department of Microbiology, Quaid-I-Azam University, Islamabad, Pakistan; f Clinical Laboratory, Meizhou People's Hospital, Meizhou, Guangdong Province, China; g Key Laboratory of Space Bioscience and Biotechnology, School of Life Sciences, Northwestern Polytechnical University, Xi’an, China; h First Clinical Medical College, Guangdong Medical University, Zhanjiang, China; i Department of Medical-Surgical and Experimental Sciences, University of Sassari Neurology Unit, Azienza Ospedaliera Universitaria (AOU) Sassari, Italy; Institut National de Santé Publique du Québec

**Keywords:** antifungal susceptibility, candidemia, candidiasis, epidemiology, risk factors

## Abstract

Candidiasis is a life-threatening disease that increases mortality in critically ill patients. However, such epidemiological data are still lacking in underdeveloped regions of China. A retrospective analysis (2016 to 2021) was conducted in Meizhou People’s Hospital, China to study the burden of candidiasis, particularly candidemia, and antifungal susceptibilities of the species among hospitalized patients. Of the 7,864 candidiasis cases, 461 (5.86%) were candidemia cases. Candida albicans (64.25%) was the most identified species, followed by C. tropicalis (12.61%), C. glabrata (10.79%), and C. parapsilosis (9.79%). In non-C. albicans (NCA) candidemia cases, the number of C. glabrata cases was higher (102/461, 22.37%) than C. tropicalis (64/461, 14.04%). Gastrointestinal pathology, respiratory dysfunctions, septic shock, and malignancies were common underlying comorbidities, respectively. A central venous catheter was an independent risk factor for both C. albicans and NCA candidemia. The mortality rate was not statistically significant for either C. albicans or NCA. Amphotericin B and 5-flucytosine were highly effective (98 to 100%), while azoles were least effective (67.74 to 95.66%). Candidemia cases caused by C. tropicalis and C. glabrata had significantly lower azole susceptibility than non-candidemia-causing isolates. This study provides valuable information for prescribers to choose the right empirical therapy, for researchers to explore different resistance mechanisms, and for health care managers to control candidiasis better.

**IMPORTANCE** This study provides important information on the burden of candidiasis, particularly candidemia, and the antifungal susceptibility of *Candida* species among hospitalized patients in an underdeveloped region of China. First, the finding that azoles were least effective against *Candida* species causing candidemia is particularly noteworthy, as it suggests the possibility of resistance to this class of antifungal agents. This information can guide the choice of empirical therapy and help in the selection of appropriate antifungal agents for the treatment of candidemia, thereby reducing the risk of resistance development. Second, the study provides important information for researchers to explore different resistance mechanisms in *Candida* species. Finally, the study has important implications for health care managers in controlling the spread of candidiasis. The high prevalence of candidemia cases in the study highlights the need for appropriate infection control measures to prevent the spread of the disease.

## INTRODUCTION

*Candida* is a nosocomial opportunistic fungal pathogen that causes severe invasive to nonfatal superficial infections in immunocompromised persons. In hospitalized patients, invasive candidiasis such as blood stream infections (candidemia) and deep-seated infections are highly reported; however, superficial, oral, and vaginal candidiasis are also well documented (1). According to the latest literature, a 7% morbidity rate has been reported globally for cutaneous candidiasis among hospitalized patients (2, 3). Similarly, annually, two and a half million people worldwide are infected with invasive candidiasis, especially with candidemia (4). The mortality rate for superficial candidiasis is relatively low; however, if the infection remains undiagnosed or untreated for a long time, it can cause invasive candidemia with a mortality rate of 25 to 50% (5). The candidemia complicates the therapeutic procedure, causing prolonged hospital stays and budget outlay, with a reported cost of $40,000 per patient (6).

To date, 40 *Candida* species have been reported to cause candidiasis in humans (7, 8). Among these, Candida albicans is reported in a high proportion worldwide. However, recent literature states the emergence of non-Candida albicans (NCA) in different regions of the world. Among the NCA, Candida glabrata, Candida tropicalis, Candida parapsilosis, and Candida krusei are the prominent members (9). Each *Candida* species has unique features, including invasive potentials, virulence and tissue tropism, and antifungal sensitivity (10). Among these, antifungal susceptibility is of special concern because of the availability of fewer antifungal agents and the emergence of multidrug resistant *Candida* pathogens (11). A recent systematic study in China reported the lowest susceptibility to azole, echinocandins, and polyenes for various *Candida* species compared to the other regions of the world. Furthermore, it stated that susceptibility profiles vary among different regions of China (9). On the other hand, the current guidelines for candidiasis management recommend empirical antifungal therapy (6). Therefore, local epidemiological and surveillance studies are needed to enhance antifungal stewardship.

This retrospective study aims to evaluate the trends in the incidence of C. albicans and NCA infections and their antifungal resistance patterns over the past 6 years in Meizhou People’s Hospital, located in Meizhou, Guangdong, China. This study will provide important information such as the distribution, risk factors, and antifungal susceptibility profiles of various *Candida* pathogens that cause candidiasis, particularly candidemia, in hospitalized patients in our locality. The outcomes of this study will assist health care officials and prescribers in managing candidiasis in the region.

## RESULTS

### Distribution of *Candida* species.

**FIG 1:**
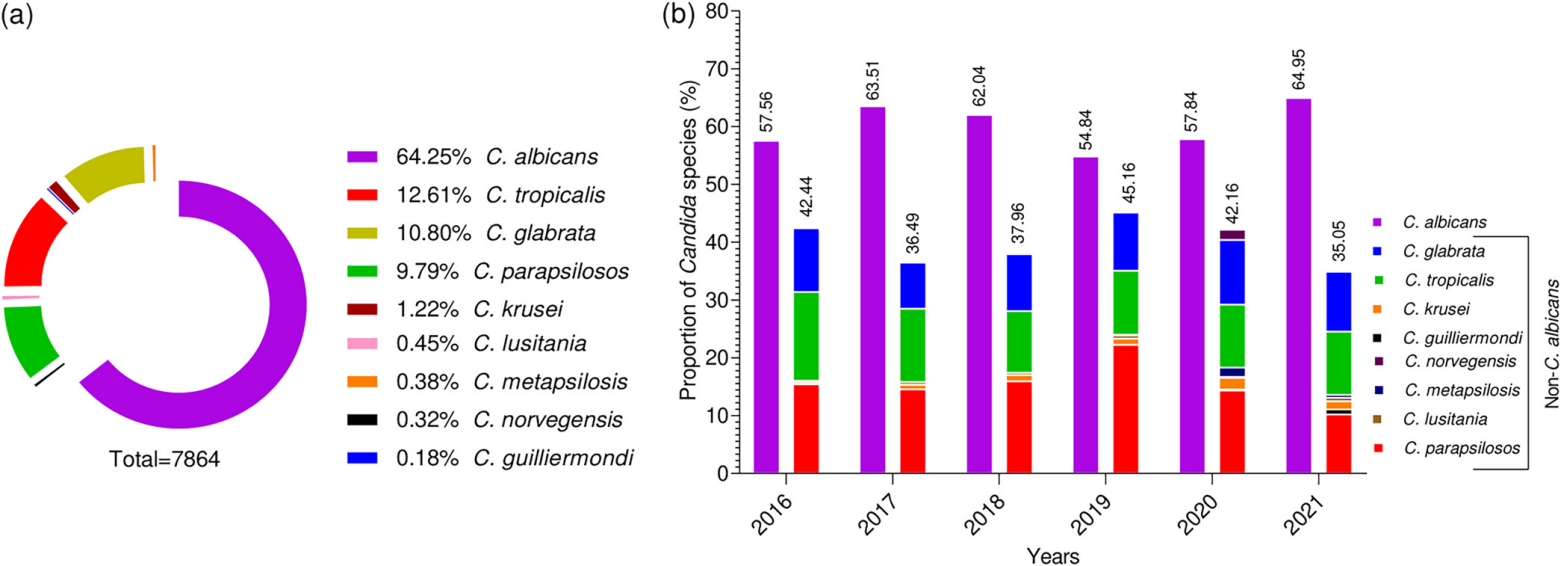
Occurrence of *Candida* species. (a) the proportion of *Candida* species detected in a 6-year study duration. (b) The annual proportion of *Candida* species reported in the current study.

**FIG 2:**
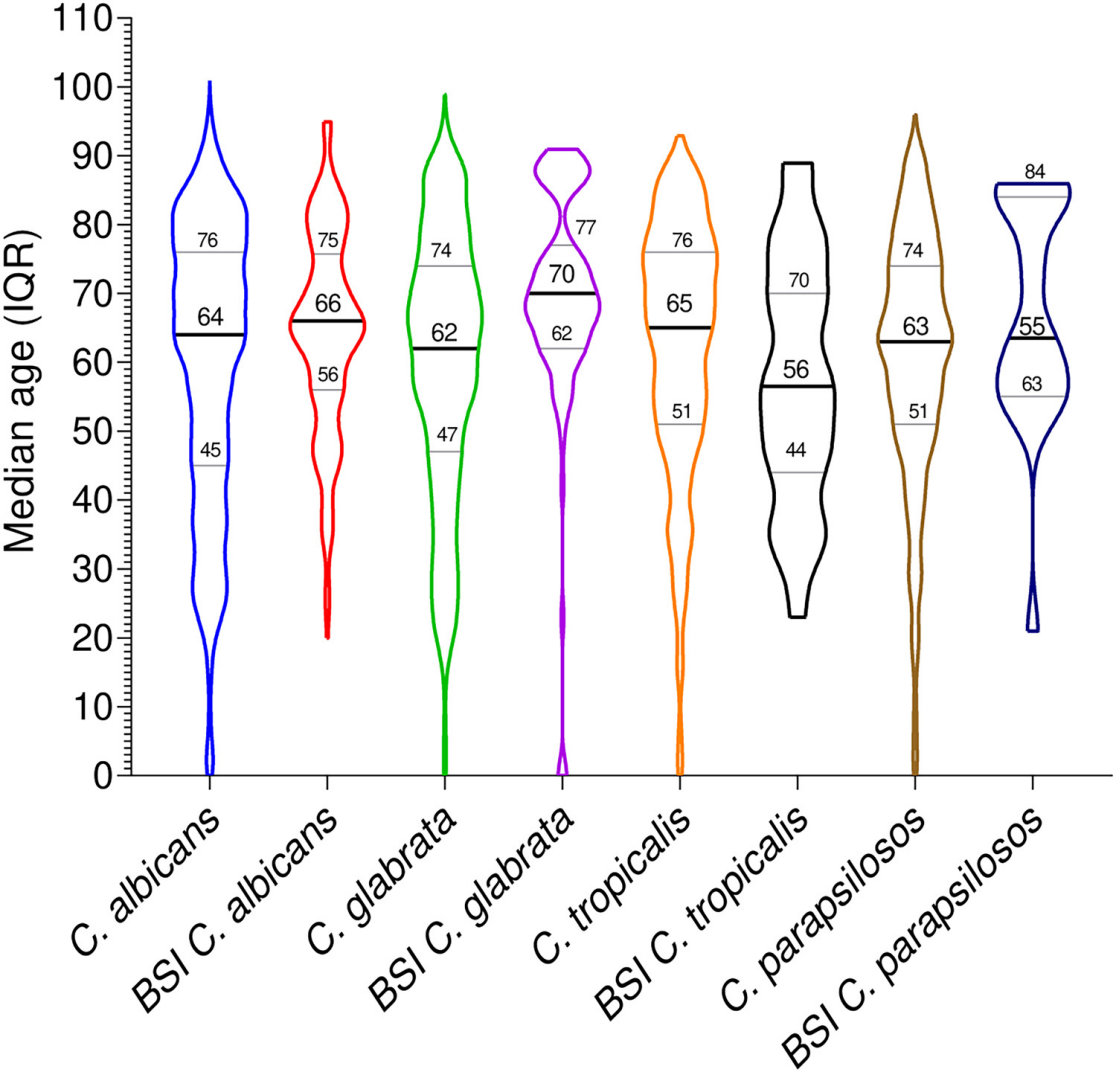
Median ages of *Candida*-infected patients. The species names represent the overall cases reported, while the BSI represents only the candidemia patients. The bold line in the middle of the violin represents the median age, while the light lines represent the IQR. The length of the violin represents the ranges of ages, while width represents the number of patients of a certain age.

During the 6-year study, 7,864 *Candida* infections were reported, containing a high proportion of C. albicans (*n *= 5,053, 64.25%), followed by C. tropicalis (*n *= 992, 12.61%) and C. glabrata (*n *= 849, 10.80%), while C. guilliermondii (*n *= 14, 0.18%) was reported with the lowest proportion. A high number of cases were reported in the year 2018 (*n *= 1,523, 19.37%), followed by 2019 (*n *= 1,486, 18.90%) and 2021 (*n *= 1,297, 16.49%). The total occurrence and annual distribution of *Candida* species are presented in Fig. 1. The median age of the patients was 64 years (interquartile range [IQR, 44 to 77]); the youngest patient was 5 days old, while the oldest was 101 years old. The median ages (IQR) of *Candida*-infected patients are presented in Fig. 2.

Regarding the age groups, *Candida* infection mainly occurred in the adult age group (*n *= 4,056, 51.57%), followed by the older patients (*n *= 3,701, 47.06%). Only 14 cases were reported in neonatal patients, of which 7 (50%) were C. albicans, and 5 (35.71%) were C. glabrata. Similarly, 24 cases were reported in infants, among which 16 (66.67%) were C. albicans and 8 (33.33%) were NCA. The number of cases in male patients (*n* = 4,146, 52.72%) was a little higher than the number of cases in female patients (*n *= 3,720, 47.3%) for overall *Candida* species, except for C. glabrata and C. norvegensis, in which the male to female ratios were 0.43:1 and 0.38:1, respectively. The mean incidence of *Candida* species per 1,000 admissions was 10.16, the highest of which was reported in 2019 (12.11/1,000), followed by 2021 (11.71/1,000), while the lowest was reported in 2017 (7.81/1,000). More specifically, for C. albicans, the mean incidence was 6.53/1,000 inpatients, the highest of which was reported in 2021 (7.93/1,000). Among the NCA, the highest mean incidence was reported for C. tropicalis (1.28/1,000), followed by C. glabrata (1.09/1,000) and C. parapsilosis (0.99/1,000). The distributions of *Candida* species based on age group and gender and annual incidence per 1,000 hospitalizations are summarized in Table 1.

**TABLE 1:** Distribution and incidence of *Candida* species

Distribution	All cases *n* = 7,864 (100%)	C. albicans *n *= 5,053 (64.25%)	C. tropicalis *n *= 992 (12.61%)	C. glabrata *n *= 849 (10.79%)	C. parapsilosis *n *= 770 (9.79%)	C. krusei *n *= 96 (1.22%)	C. lusitaniae *n *= 35 (0.44%)	C. metapsilosis *n *= 30 (0.38%)	C. norvegensis *n *= 25 (0.31%)	C. guilliermondii *n *= 14 (0.17%)
Age group [*n* (%)]										
Neonatal	14 (0.17)	7 (50)	0 (0.00)	5 (35.71)	1 (7.14)	1 (7.14)	0 (0.00)	0 (0.00)	0 (0.00)	0 (0.00)
Infants	24 (0.3)	16 (66.66)	4 (16.66)	1 (4.16)	2 (8.33)	0 (0.00)	0 (0.00)	0 (0.00)	0 (0)	1 (4.16)
Children	69 (0.87)	8 (11.59)	26 (37.68)	12 (17.39)	22 (31.88)	1 (1.44)	0 (0.00)	0 (0.00)	0 (0.00)	0 (0.00)
Adults	4,056 (51.57)	2,540 (62.62)	494 (12.17)	491 (12.1)	434 (10.7)	55 (1.35)	14 (0.34)	16 (0.39)	5 (0.12)	7 (0.17)
Older	3,701 (47.06)	2,482 (67.06)	468 (12.64)	340 (9.18)	311 (8.4)	39 (1.05)	21 (0.56)	14 (0.37)	20 (0.54)	6 (0.16)
Gender [*n* (%)]										
Male	4,146 (52.72)	2,698 (65.07)	556 (13.41)	256 (6.17)	517 (12.46)	59 (1.42)	21 (0.5)	19 (0.45)	7 (0.16)	13 (0.31)
Female	3,720 (47.3)	2,355 (63.3)	436 (11.72)	593 (15.94)	255 (6.85)	37 (0.99)	14 (0.37)	11 (0.29)	18 (0.48)	1 (0.02)
Male: Female	1.11:1	1.14:1	1.27:1	0.43:1	2.02:1	1.59:1	1.5:1	1.72:1	0.38:1	13:01
Incidence/1,000 inpatients										
2016	8.47	5.12	1.36	0.98	0.94	0.03	0.02	0.00	0.00	0.00
2017	7.81	5.23	1.04	0.65	0.77	0.06	0.03	0.00	0.00	0.00
2018	11.21	7.54	1.29	1.2	0.98	0.13	0.05	0.00	0.00	0.00
2019	12.11	7.43	1.49	1.36	1.57	0.14	0.07	0.01	0.00	0.00
2020	10.41	6.36	1.19	1.23	0.98	0.22	0.01	0.17	0.19	0.01
2021	11.71	7.93	1.33	1.26	0.74	0.17	0.07	0.06	0.01	0.1
Mean	10.16	6.53	1.28	1.09	0.99	0.12	0.04	0.03	0.03	0.01

**FIG 3:**
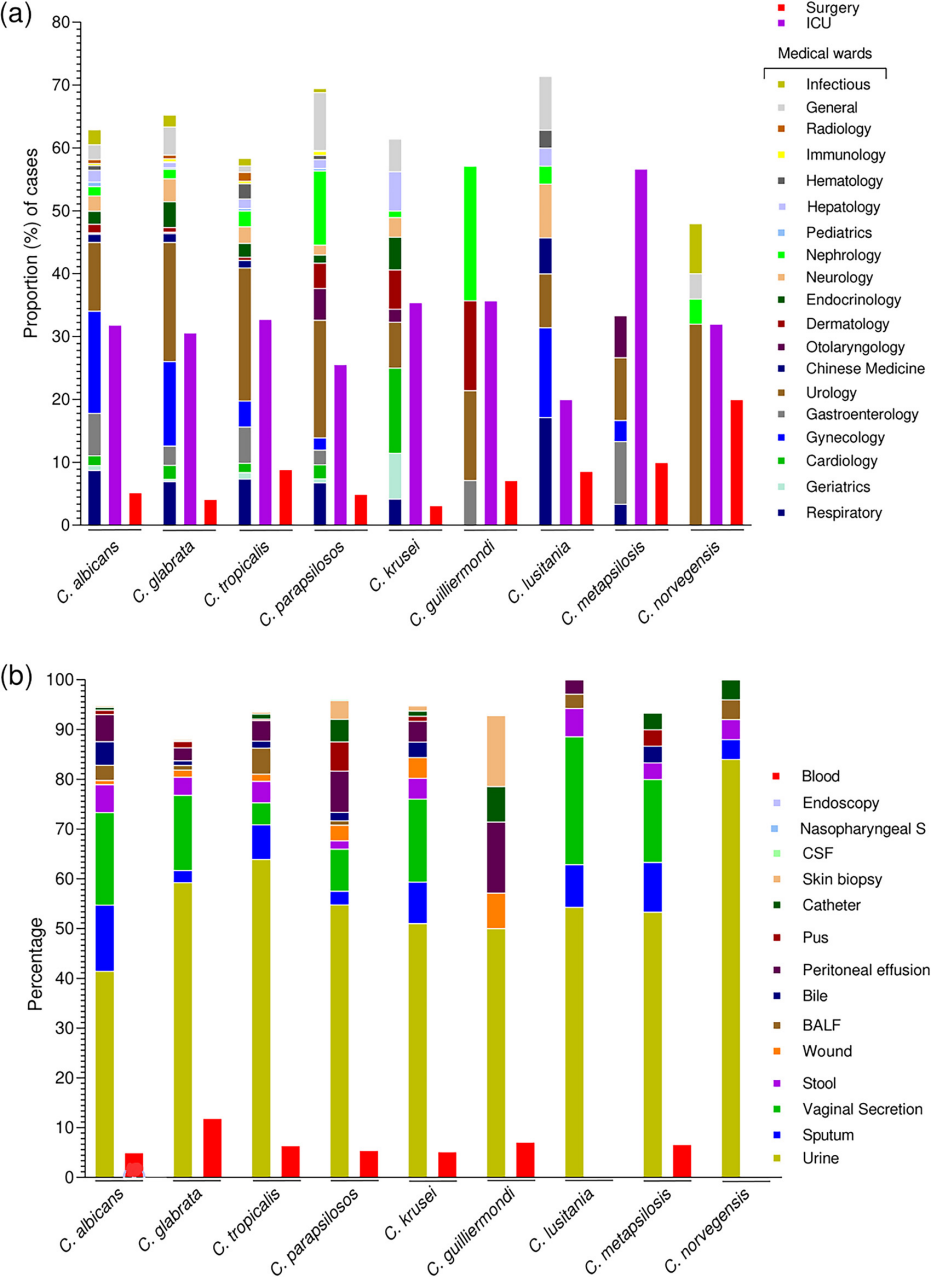
Proportion of *Candida* species from hospital wards and sample sources. (a) Percentages of *Candida* species reported from the various departments of the hospital. (b) Proportion of *Candida* species isolated from various sample sources. BALF, bronchoalveolar lavage fluid.

Among the hospital departments, a high number of cases were reported from the intensive care unit (ICU) (*n* = 2,463, 31.32%), while 439 (5.58%) cases were reported from the surgical department. The proportions of *Candida* species reported from different departments of the hospital is presented in Fig 3a. Among the sample sources, a high proportion of cases was reported in urine (*n* = 3,765, 47.87%), followed by vaginal secretion (*n* = 1,208, 15.36%) and sputum (*n* = 798, 10.14%), while a total of 456 (5.80%) cases were reported from blood samples. The proportion of *Candida* species isolated from various sample sources is depicted in Fig. 3b.

### Clinical characteristics associated with C. albicans and non-C. albicans.

The baseline clinical characteristics associated with C. albicans and NCA are summarized in Table 2. Overall, 47.06% of the patients belonged to the older age group, in which the proportion for C. albicans was higher than that for NCA, and their differences were statistically significant (*P* < 0.0001). The male-to-female infection ratios of C. albicans and NCA were not significant. For the underlying comorbidities cases, a high proportion was enumerated for respiratory dysfunction (12.51%), followed by renal failure (8.59%), urinary tract infection (UTI) (7.21%), neurological diseases (6.86%), and gastrointestinal pathology (6.14%). The proportion of severity of tuberculosis, solid tumors, diabetes, and cardiovascular diseases was statistically insignificant among the cases of C. albicans and NCA. Moreover, we noted that the proportions of some comorbidities, such as renal failure, digestive tract pathology, UTI, neurological diseases, and otitis media were higher for NCA than C. albicans cases, and their differences were statistically significant (*P < *0.05). Among the prior invasive procedures, a high proportion of cases were reported in association with urinary tract catheters (11.86%), followed by mechanical ventilation (9.72%) and parenteral nutrition (8.35%). Surprisingly, the proportion of all previous invasive procedures was high for NCA than C. albicans (*P < *0.05). The median time of hospital stays of patients infected with NCA was 29 days (IQR, 15 to 41), and for C. albicans it was 24 days (IQR, 10 to 36). No statistical differences were noted for C. albicans and NCA for the patients that previously stayed in the ICU. The proportions of 7-day mortality for C. albicans (4.61%) and NCA (5.08%) were statistically not significant. However, the 30-day and all-cause mortality rates for NCA (22.27% and 26.39%, respectively) were high in the patients infected with C. albicans (18.99% and 22.99%, respectively [*P < *0.05]).

**TABLE 2:** Univariant analysis of demographic data and clinical characteristics of all patients infected with C. albicans versus non-C. albicans

Variable	Total [*n* (%)] (7,864, 100%)	C. albicans [*n* (%)] (5,053, 64.25%)	NCA^*a*^ [*n* (%)] (2,811, 35.74%)	Z value	*P* value
Age > 65	3,701 (47.06)	2,482 (49.11)	1,219 (43.36)	4.899	<0.0001
Male:female	4,146:3,720	2,698:2,355	1,448:1,365	0.8653^*b*^	0.2393^*c*^
Underlying conditions					
Gastrointestinal pathology	483 (6.14)	245 (4.84)	238 (8.46)	6.404	<0.0001
Renal failure	676 (8.59)	275 (5.44)	401 (14.26)	13.38	<0.0001
Respiratory dysfunction	984 (12.51)	661 (13.08)	323 (11.49)	2.043	0.041
Tuberculosis	29 (0.36)	20 (0.39)	9 (0.32)	0.5303	0.5959
Liver diseases	257 (3.26)	189 (3.74)	68 (2.41)	3.158	0.0016
Hematologic malignancy	252 (3.2)	140 (2.77)	112 (3.98)	2.929	0.0034
UTI	567 (7.21)	303 (5.99)	264 (9.39)	5.579	<0.0001
Solid tumor	296 (3.76)	187 (3.7)	109 (3.87)	0.3949	0.6929
Neurological diseases	540 (6.86)	316 (6.25)	224 (7.96)	2.882	0.0039
Diabetes	213 (2.7)	133 (2.63)	80 (2.84)	0.5599	0.5755
Cardiovascular diseases	337 (4.28)	217 (4.29)	120 (4.26)	0.05358	0.9573
Fever (Tm ≥ 38°C)	366 (4.65)	219 (4.33)	147 (5.22)	1.806	0.0708
Compromised skin barrier	330 (4.19)	250 (4.94)	80 (2.84)	4.455	<0.0001
Otitis media	44 (0.55)	10 (0.19)	34 (1.2)	5.764	<0.0001
Pregnancy	207 (2.63)	158 (3.12)	49 (1.74)	3.673	0.0002
Hypertension	86 (1.09)	67 (1.32)	19 (0.67)	2.656	0.0079
Bone diseases	41 (0.52)	22 (0.43)	19 (0.67)	1.419	0.1558
Previous invasive procedures					
Urinary tract catheter	933 (11.86)	543 (10.74)	390 (13.87)	4.111	<0.0001
Central venous catheter	310 (3.94)	140 (2.77)	170 (6.04)	7.157	<0.0001
Mechanical ventilation	765 (9.72)	364 (7.2)	401 (14.26)	10.13	<0.0001
Parenteral nutrition	657 (8.35)	350 (6.92)	307 (10.92)	6.136	<0.0001
Surgery	439 (5.58)	263 (5.2)	176 (6.26)	1.955	0.0505
Hospital stay (days)^*d*^	26 (12–40)	24 (10–36)	29 (15–41)		
Previous ICU stay	2,463 (31.31)	1,610 (31.86)	853 (30.34)	1.39	0.1644
Mortality					
7-day mortality	376 (4.78)	233 (4.61)	143 (5.08)	0.9482	0.343
30-day mortality	1,589 (20.2)	960 (18.99)	629 (22.37)	3.575	0.0004
All-cause mortality	1,904 (24.21)	1,162 (22.99)	742 (26.39)	3.373	0.0007

a Non-C. albicans.

b R squared value.

c Ratio paired *t* test.

d Median (IQR).

### Candidemia.

Furthermore, the data for *Candida* species reported from blood samples were retrieved to analyze the epidemiology of candidemia. A total of 461 candidemia cases were noted, of which 245 (53.72%) were C. albicans, 102 (22.37%) were C. glabrata, 64 (14.04%) were C. tropicalis, 42 (9.21%) were C. parapsilosis, 5 (1.10%) were C. krusei, 2 (0.44%) were C. metapsilosis, and 1 (0.22%) was C. guilliermondii. The median age of patients was 67 years (IQR, 58 to 78); the youngest was 20 years old, while the oldest was 91 years old. Among the various age groups, almost half of the patients (*n =* 228, 49.46%) were from the older age group. The median age of candidemia patients infected by various *Candida* species is presented in Fig. 2. The proportion of male cases was high compared to that of females, at the ratio of 3.15:1; which C. albicans the ratio was 3.08:1, and for NCA it was 3.23:1. Among different hospital departments, a large number of cases were reported from the ICU (*n* = 306, 66.37%), followed by medical wards (*n* = 106, 22.99%), while 49 (10.62%) cases occurred in the surgical department. In ICU cases, 170 (47.22%) were C. albicans, 83 (27.12%) were C. glabrata, 28 (9.15%) were C. tropicalis, 21 (6.86%) were C. parapsilosis, 2 (0.65%) were C. krusei, and 2 (0.65%) were C. metapsilosis. The clinical characteristics associated with candidemia due to C. albicans and non-C. albicans are presented in Table 3. Among the reported underlying conditions, many patients suffered from gastrointestinal pathology (15.83%), followed by respiratory dysfunctions (9.76%), septic shock (6.94%), and malignancies (5.21%). Moreover, we noted that the proportion of gastrointestinal pathology, cardiovascular diseases, and septic shock was significantly higher in non-C. albicans cases than in C. albicans (*P < *0.05). However, respiratory dysfunction, solid tumor, and hypertension were high in C. albicans cases compared to non-C. albicans. Among the prior invasive procedures, a high proportion of cases was reported in association with mechanical ventilation (52.28%), followed by central venous catheter (49.02%), urinary tract catheter (45.53%), and parenteral nutrition (41.21). Furthermore, we found a statistically significant association with C. albicans and NCA (*P < *0.05). The 7-day, 30-day, and all-cause mortality rates for candidemia patients were 29 (6.2%), 83 (18%), and 91 (19.74%), respectively, and were not statistically different for C. albicans versus NCA (*P* > 0.05).

**TABLE 3:** Univariant analysis of demographic data and clinical characteristics of candidemia patients infected with C. albicans versus non-C. albicans

Variable	Total (*n* [%]) (461, (100%)	C. albicans (*n* [%]) (245, 53.14%)	NCA^*a*^ [*n* (%)] (216, 46.86%)	Z value	*P* value
Age > 65	228 (49.46)	124 (50.61)	104 (49.28)	0.5280	0.5975
Male:female	350:111	185:60	165:51	0.9996^*b*^	0.0133^*c*^
Underlying conditions					
Gastrointestinal pathology	73 (15.83)	27 (11.02)	46 (21.30)	3.016	0.0026
Renal failure	9 (1.95)	5 (2.04)	4 (1.85)	0.1463	0.8837
Respiratory dysfunction	45 (9.76)	32 (13.06)	13 (6.02)	2.542	0.0110
Liver diseases	8 (1.73)	6 (2.44)	2 (0.93)	1.250	0.2114
Hematologic malignancy	22 (4.77)	8 (3.26)	14 (6.48)	1.616	0.1060
UTI	8 (1.73)	4 (1.63)	4 (1.85)	0.1798	0.8573
Solid tumor	24 (5.21)	18 (7.34)	6 (2.78)	2.204	0.0275
Neurological diseases	22 (4.77)	13 (5.3)	9 (4.17)	0.5727	0.5669
Diabetes	9 (1.95)	3 (1.22)	6 (2.78)	1.203	0.2290
Cardiovascular disease	23 (4.99)	8 (3.26)	15 (6.94)	1.811	0.0702
Septic shock	32 (6.94)	8 (3.26)	24 (11.11)	3.307	0.0009
Compromised skin barrier	5 (1.08)	5 (2.04)	0 (0.00)	2.111	0.0348
Pregnancy	7 (1.51)	4 (1.63)	3 (1.38)	0.2136	0.8309
Hypertension	11 (2.39)	9 (3.67)	2 (0.93)	1.929	0.0537
Previous invasive procedure					
Urinary tract catheter	210 (45.53)	97 (39.59)	113 (52.31)	2.737	0.0062
Central venous catheter	226 (49.02)	105 (42.85)	121 (56.02)	2.821	0.0048
Mechanical ventilation	241 (52.28)	114 (46.53)	127 (58.79)	2.631	0.0085
Parenteral nutrition	190 (41.21)	87 (35.51)	103 (47.68)	2.650	0.0080
Surgery	49 (10.62)	31 (12.65)	18 (8.33)	1.502	0.1332
Hospital stay (days)^*d*^	30 (16–42)	26 (18–39)	32 (12–44)		
Previous ICU stay	306 (66.37)	170 (69.38)	136 (62.96)	1.457	0.1451
Mortality					
7-day mortality	29 (6.29)	13 (5.3)	16 (7.41)	0.9273	0.3538
30-day mortality	83 (18.00)	49 (20)	34 (15.74)	1.188	0.2349
All-cause mortality	91 (19.74)	52 (21.22)	39 (18.06)	0.8530	0.3937

a Non-C. albicans.

b R squared value;

c Ratio paired *t* test.

d Median (IQR).

Furthermore, the odds ratios (95% confidence interval [CI]) for independent risk factors of candidemia due to C. albicans and NCA were found using the Baptista-Pike method. Among all factors, the odds ratio for central venous catheter was greater for C. albicans (3.042; 95% CI, 2.067 to 4.481) than for NCA – (2.535; 95% CI, 1.820 to 3.542). For all other risk factors, the odd ratios were, as shown in Table 4.

**TABLE 4:** Odd ratios of risk factors associated with candidemia caused by C. albicans and non-C. albicans

Variable	C. albicans			Non-C. albicans		
	Odds ratio	95% CI	*P* value	Odds ratio	95% CI	*P* value
Age > 65 yrs	0.028	0.024–0.035	<1 × 10^−15^	0.05	0.040–0.061	<1 × 10^−15^
Previous ICU stay	0.087	0.074–0.103	<1 × 10^−15^	0.148	0.122–0.178	<1 × 10^−15^
Central venous catheter	3.042	2.067–4.481	2.5 × 10^−9^	2.535	1.820–3.542	2 × 10^−8^
Parenteral nutrition	0.319	0.250–0.408	<1 × 10^−15^	0.486	0.382–0.621	3 × 10^−9^
Urinary tract catheter	0.202	0.162–0.253	<1 × 10^−15^	0.383	0.305–0.478	<1 × 10^−15^
Mechanical ventilation	0.443	0.353–0.553	3.8 × 10^−13^	0.438	0.352–0.543	2.6 × 10^−14^
Surgery	0.128	0.088–0.186	<1 × 10^−15^	0.108	0.065–0.176	<1 × 10^−15^
Gastrointestinal pathology	0.062	0.043–0.092	<1 × 10^−15^	0.122	0.089–0.166	<1 × 10^−15^
Pulmonary dysfunction	0.044	0.031–0.064	<1 × 10^−15^	0.013	0.008–0.024	<1 × 10^−15^
Liver diseases	0.031	0.015–0.070	<1 × 10^−15^	0.026	0.006–0.098	<1 × 10^−15^
Hematologic malignancy	0.059	0.028–0.117	<1 × 10^−15^	0.088	0.051–0.150	<1 × 10^−15^
Diabetes	0.022	0.007–0.066	<1 × 10^−15^	0.073	0.035–0.167	<1 × 10^−15^
Cardiovascular diseases	0.037	0.017–0.073	<1 × 10^−15^	0.08	0.048–0.136	<1 × 10^−15^
Septic shock	0.036	0.017–0.072	<1 × 10^−15^	0.221	0.141–0.342	5.4 × 10^−13^

### Antifungal susceptibility profiles.

The antifungal susceptibility profiles of all *Candida* species reported in the current study are summarized in Table 5. Among the five tested antifungal drugs, the highest susceptibilities were reported for amphotericin B and 5-flucytosine against all *Candida* species. For C. albicans, the susceptibilities against fluconazole, voriconazole, and itraconazole were 92.06%, 90.37%, and 78.71%, respectively. Among the NCA, C. parapsilosis, and C. guilliermondii were the most susceptible, as the proportion of susceptible/wild-type isolates was greater than 95% against all five tested antifungal agents. For C. tropicalis, C. glabrata, and C. krusei, the lowest susceptibilities were reported against itraconazole: 82.88%, 86.78%, and 94.37%, respectively.

**TABLE 5:** Antifungal susceptibility profiles of *Candida* isolates in the current study

*Candida* spp.	Antifungal agent	Tested organisms (*n*)	S/WT [*n* (%)]^*a*^	SDD/I [*n* (%)]^*b*^	R/nWT [*n* (%)]^*c*^
C. albicans	Amphotericin B	4,322	4,317 (99.88)		5 (0.12)
	5-Flucytosine	1,375	1,355 (98.55)		20 (1.45)
	Fluconazole	4,649	4,280 (92.06)	186 (4.00)	183 (3.94)
	Itraconazole	3,063	2,411 (78.71)	551 (17.99)	101 (3.30)
	Voriconazole	4,590	4,148 (90.37)	310 (6.75)	132 (2.88)
C. tropicalis	Amphotericin B	872	871 (99.88)		1 (0.12)
	5-Flucytosine	258	258 (100)		0
	Fluconazole	928	797 (85.88)	18 (1.94)	113 (12.18)
	Itraconazole	771	639 (82.88)		132 (17.12)
	Voriconazole	933	794 (85.10)	46 (4.93)	93 (9.97)
C. glabrata	Amphotericin B	698	697 (99.86)		1 (0.14)
	5-Flucytosine	246	246 (100)		0
	Fluconazole	735		513 (69.80)	222 (30.20)
	Itraconazole	605	525 (86.78)		80 (13.22)
	Voriconazole	760	676 (88.95)		84 (11.05)
C. parapsilosis	Amphotericin B	682	676 (99.12)		6 (0.88)
	5-Flucytosine	219	218 (99.54)		1 (0.46)
	Fluconazole	738	706 (95.66)	9 (1.22)	23 (3.12)
	Itraconazole	586	572 (97.61)		14 (2.39)
	Voriconazole	738	727 (98.51)	6 (0.81)	5 (0.68)
C. krusei	Amphotericin B	77	77 (100)		0
	5-Flucytosine	42	40 (95.24)		2 (4.76)
	Itraconazole	71	67 (94.37)		4 (5.63)
	Voriconazole	87	86 (98.85)	1 (1.15)	0
C. guilliermondii	Amphotericin B	12	12 (100)		0
	5-Flucytosine	12	12 (100)		0
	Fluconazole	12	12 (100)	0	0
	Itraconazole	12	12 (100)		0
	Voriconazole	12	12 (100)	0	0
C. lusitaniae	Amphotericin B	29	29 (100)		0
	5-Flucytosine	8	8 (100)		0
	Fluconazole	31	21 (67.74)	2 (6.45)	8 (25.81)
	Itraconazole	24	18 (75)		6 (25)
	Voriconazole	28	22 (78.57)	2 (7.14)	4 (14.29)
C. metapsilosis	Amphotericin B	25	23 (92)		2 (8)
	5-Flucytosine	28	26 (92.86)		2 (7.14)
	Fluconazole	28	26 (92.86)	2 (7.14)	0
	Itraconazole	28	26 (92.86)		2 (7.14)
	Voriconazole	28	26 (92.86)	2 (7.14)	0
C. norvegensis	Amphotericin B	24	24 (100)		0
	5-Flucytosine	24	21 (87.5)		3 (12.5)
	Fluconazole	24	19 (79.17)	4 (16.67)	1 (4.16)
	Itraconazole	24	19 (79.17)		5 (20.83)
	Voriconazole	24	24 (100)	0	0

a Susceptible or wild type.

b Susceptible dose dependent or intermediate.

c Resistant or non-wild type.

**FIG 4:**
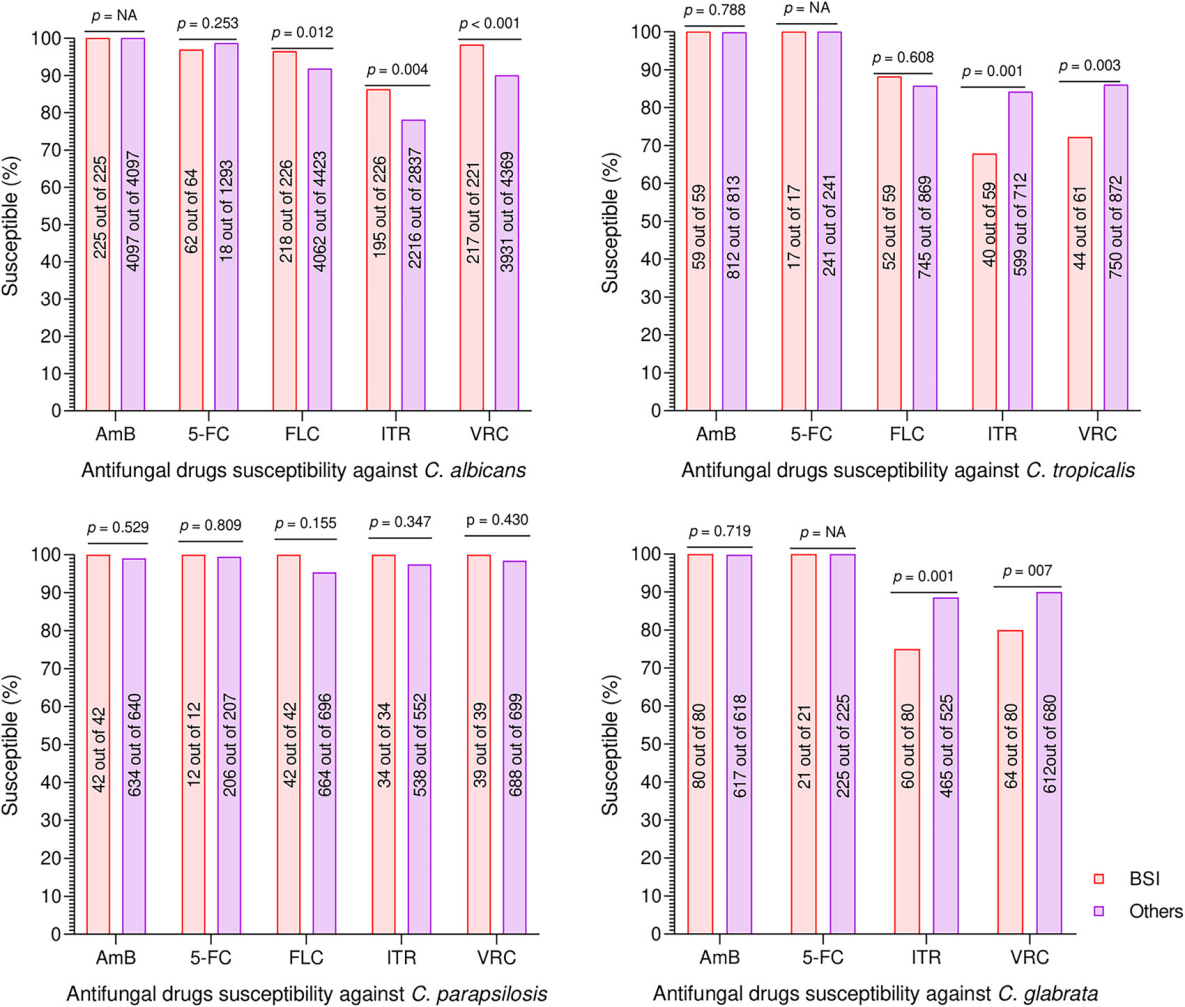
Comparison of susceptibilities/wild types of isolates recovered from candidemia (BSI) cases versus nonbloodstream *Candida* infections (other). The pink bars indicate candidemia (BSI) isolates, while the purple bars show nonbloodstream infections. *P* values of <0.05 are statistically significant. AmB, amphotericin B; 5-FC, 5-flucytosine; FLC, fluconazole; ITR, itraconazole; VRC, voriconazole.

Furthermore, we compared the susceptibility profiles of *Candida* species recovered from candidemia cases with the isolates recovered from nonbloodstream *Candida* infections (Fig. 4). The susceptibilities of amphotericin B and 5- flucytosine were statistically not different in all tested isolates in both groups. Similarly, for C. parapsilosis, the susceptibilities against all five tested drugs were not statistically significant (*P > *0.05). Interestingly, in the case of C. albicans, the susceptibilities of all three tested azole drugs were high for candidemia compared to the other group (*P < *0.05). In contrast, in C. tropicalis and C. glabrata, the susceptibility against itraconazole and voriconazole of candidemia-causing isolates was lower than in the other group (*P < *0.05). However, the fluconazole susceptibility of C. tropicalis in the two groups was not statistically different (*P > *0.05).

## DISCUSSION

In the present study, we retrospectively analyzed the occurrence, risk factors, and antifungal susceptibility profiles of *Candida* pathogens in hospitalized patients from a tertiary care hospital in Meizhou, China. This study will provide information for health care providers to manage candidiasis better.

In our study, C. albicans was detected in a high proportion compared to NCA. This contrasts with some recent reports that mentioned the emergence of a high proportion of NCA (12). However, our study is in line with some previous reports from China that had abundant C. albicans instead of NCA (13). The epidemiological trends of C. albicans and NCA vary substantially depending on geography, diagnostic center, and type of patients (4, 14). Among the NCA, a high proportion was reported for C. glabrata, followed by C. tropicalis, while in the candidemia cases, the proportion of C. tropicalis was higher than that of C. glabrata. As in our study, a high proportion of C. glabrata among NCA was previously reported in North America and many European countries (15). However, studies from India, Cameroon, and Nigeria reported a high proportion of C. tropicalis compared to C. glabrata (16). Specifically, in candidemia cases, a high proportion of C. tropicalis, like our study, is also reported from other regions of China with similar latitudes to ours (13, 17). However, in the studies from different-latitude regions, such as North America, western Europe, and northern China, C. glabrata is reported in high proportions in candidemia patients (18–20). A study from Thailand stated that C. tropicalis is an exogenous isolate acquired from environments (21).

Regarding gender, a slightly higher number of cases were reported in the male population than in females. Some other studies from China reported a similar distribution (22, 23); however, a study from Poland reported almost double the number of females with candidiasis (24). Differences in candidiasis incidence between males and females can be attributed to anatomical and physiological differences between the sexes. The higher incidence of candidiasis in males in China might be due to differences in health care practices or cultural attitudes toward the condition (25). However, no specific reason is available in the published literature, and further study is needed to investigate the possible reasons. The median age of the patients with candidiasis was 60 to 70 years for different *Candida* pathogens, which is also similar to other studies (26–29). These aged patients are more vulnerable to infections due to their immunocompromised status and underlying critical sickness (30). The highest number of patients were reported from ICUs, aligned with the previously published study from China (13, 31). The ICU patients are mostly in critical condition and immunocompromised, allowing nosocomial *Candida* to become pathogenic (31–33). Moreover, the use of invasive procedures such as central venous catheters, urinary tract catheters, mechanical ventilation, and parenteral nutrition is high in ICU patients, providing a path for *Candida* pathogenesis (34, 35). A total of 439 (5.58%) cases were reported from the surgical department. Surgery is considered a secondary risk factor for nosocomial dissemination of candidiasis (9).

Similarly, central venous catheters (CVC) were found to be an independent risk factor for both C. albicans and NCA, with an odds ratio greater than 1 in the current study. A similar study from Europe (Croatia) reported CVC as an independent risk factor of invasive candidiasis (36). It is well-known most *Candida* species inhabit the hand skin and can colonize on catheters. This suggests that a wide range of invasive candidiasis might be due to the horizontal transmission of *Candida* species by health care workers (37). Hospital staff should pay special attention to hand washing and decontamination to minimize the risk of horizontal transmission of nosocomial candidiasis (9). In overall cases, a high number of *Candida* species were recovered from urine samples. The *Candida* species reported in urine samples characterize several situations needing vigilant interpretation, including nosocomial candidiasis or contamination of samples to urinary tract infections (38, 39).

Among the underlying comorbidities, respiratory dysfunction, digestive tract pathology, and renal failure were reported in high proportions, similar to previously published literature from China (6, 22, 23, 31). The proportion of hematological malignancies for NCA candidemia was high compared to that for C. albicans in the present study. This finding is similar to the previously reported study, and it might be due to the immunosuppression induced by cytotoxic chemotherapy (13).

Several studies reported discrepant results regarding the mortality rates of C. albicans and NCA infections. In the present study, the differences in the 7-day mortality rate for overall candidiasis and 7-day, 30-day, and all-cause mortality rates for candidemia cases were statistically insignificant in C. albicans compared to NCA. Similar to our findings, previously published literature from the United States and Beijing and Shanghai, China, reported statistically insignificant differences in mortality rates in C. albicans compared to NCA infections (40–42), although in contrast to our investigation, some other studies reported significantly higher mortality rates for either C. albicans or NCA. Studies from Shenyang, Nanjing, and Shandong, China, demonstrated significantly higher mortality rates for C. albicans infection (20, 43, 44). On the other hand, studies from Greece and Shanghai, China, reported significantly higher mortality rates for NCA infections (42, 45). In our study, the statistically insignificant difference in the mortality rate for C. albicans and NCA might be due to reported nonsignificant differences in most underlying diseases for both types of infections. It is well known that mortality associated with *Candida* infection is not solely attributable to the pathogenicity of the *Candida* species, but also to a failure of host defense mechanisms and complications associated with the patient’s underlying disease (20). Though the mortality rate for both types of infection in our study is statistically insignificant, still the percentage of 7-day mortality for NCA (7.41%) was higher than that of C. albicans (5.43%). This may be due to the previously used CVC, which was more prevalent in NCA (56.02%) than in C. albicans (42.85%) cases. Similarly, the late mortality percentages were higher for C. albicans infection than for NCA cases. This might be linked with organ dysfunction and underlying diseases, as percentages of renal failure (2.05% versus 1.85%) and respiratory dysfunction (13.06% versus 6.02%) were higher in C. albicans cases than NCA infections. According to the available guidelines and published literature, early mortality is reduced due to prompt therapeutic measures, such as the early removal of intravascular catheters and proper antifungal therapy (34, 46). In contrast, late mortality is linked to host factors such as signs of organ dysfunction and patients’ comorbidity status (13).

Among the tested antifungal agents, amphotericin B and 5-flucytosine showed high susceptibility rates toward the tested pathogens. The high susceptibility to amphotericin B is mainly due to its less frequent use, as it causes severe renal toxicity and is economically unsuitable (9, 47–49). For 5-flucytosine, 20 C. albicans and 8 NCA isolates showed resistance. Nonetheless, these isolates did not exhibit any cross-resistance to other antifungal agents, similar to previously reported studies from Brazil and China (50, 51). Comparatively low susceptibilities were reported against azole drugs; against C. albicans, the itraconazole, voriconazole, and fluconazole susceptibility rates were 78.71%, 90.37%, and 92.06%, respectively. Likewise, a relatively low susceptibility rate for NCA against azoles was detected (82 to 89% of the isolates). Worldwide, low susceptibility of azoles against *Candida* species was reported; in several European countries, Australia, and the United States, 80 to 90% of C. glabrata isolates were azole susceptible (1, 52, 53). Similarly, nearly 90% of C. tropicalis isolates from Chile, Latin America, and the Asia Pacific region were azole susceptible (1, 53, 54). The low susceptibility against azoles might be due to their easy availability, widespread prescriptions, and agricultural usage, which leads to molecular alteration of ergosterol biosynthetic pathways, causing azole resistance. Also, we found cross-resistance among the three azole drugs, which might be related to increased efflux pump activity or Erg11p alterations (55). Among the azole drugs, the resistance against itraconazole was high compared to that against fluconazole and voriconazole. These findings are inconsistent with another study from southwest China (56). The high resistance rate of itraconazole compared to fluconazole and voriconazole might be due to its broader spectrum of activity, higher dosage requirements, and different mechanisms of action from other triazoles (57–59).

Furthermore, we compared the susceptibility profiles of candidemia and noncandidemia isolates. For C. albicans, the candidemia-causing isolates were more susceptible to azole drugs than noncandidemia isolates. For C. parapsilosis, no statistical significance was recorded among the susceptibility profiles, while for C. tropicalis and C. glabrata, the voriconazole and itraconazole susceptibilities of candidemia-causing isolates were lower than those of the non-candidemia-causing isolates. The lower susceptibilities of candidemia-causing NCA isolates might be due to prescribers’ persisting use of empirical prophylactic treatments (6). Additionally, this study shows that NCA was more prone to becoming resistant than C. albicans (60). Based on our findings, we recommend conducting additional research to determine the appropriate use of azole drugs as empirical therapy (21, 61).

The limitation of the current study is that it is a single-center study, and due to its retrospective nature, some factors for analysis were unavailable. Furthermore, antifungal susceptibility testing (AFST) was performed with an ATB FUNGUS 3 kit; hence, the echinocandins and posaconazole data were unavailable. Therefore, the results may not apply to all settings in China.

Future research is required to concentrate on the molecular mechanisms of antifungal resistance in various *Candida* species. Such studies can help identify novel antifungal therapy targets and enhance the efficacy of existing drugs. Multicenter studies could address the limitation of a single center, while prospective studies could provide more robust data. Continuous surveillance studies, advancements in rapid noncultured diagnostic approaches, and antifungal stewardship are required to halt the antifungal drug resistance issue (11). Additionally, studies are required to determine the connection between candidemia and central venous catheterization. The research in this area could help identify strategies for preventing candidemia caused by catheters. Furthermore, researchers can investigate the use of combination therapy in treating candidemia, especially in cases caused by NCA species with decreased susceptibility to azoles. Lastly, studies are required to investigate the prevalence of candidiasis in other underdeveloped regions and the epidemiological differences between regions. Such research can aid in the identification of region-specific strategies for the prevention and treatment of candidiasis.

**Conclusion.** In the current study, we retrospectively analyzed the distribution, risk factors, and antifungal susceptibility pattern of the *Candida* pathogen in Meizhou, China. C. albicans isolates were found in a high ratio, followed by C. tropicalis, while in candidemia patients, C. glabrata was more frequent than C. tropicalis. Non-C. albicans candidemia was most common in patients with gastrointestinal disorders, hematological malignancy, septic shock and those who used prior invasive procedures. The central venous catheter was an independent risk factor for both C. albicans and NCA causing candidemia. Amphotericin B and 5-flucytosine were highly active drugs, while low susceptibility was reported against azoles. For C. tropicalis and C. glabrata, the isolates causing candidemia had significantly lower azole susceptibility than non-candidemia-causing isolates. Further molecular investigations for the in-depth analysis of azole resistance magnitudes and continuous surveillance studies are required.

## MATERIALS AND METHODS

### Study design and setting.

The current 6-year (2016 to 2021) retrospective study was conducted at the 1,000-bed tertiary (A) Meizhou People’s Hospital located in Meizhou, which provides health services to 20 million people in the Guangdong province, China. The diagnosis of *Candida* infections was based on the guidelines of the China Medical Association and the Infectious Diseases Society of America for candidiasis management (34, 62). The demographic data and clinical characteristics of all inpatients with candidiasis were collected from the electronic medical records of the hospital. The data were analyzed according to patients’ age, gender, sample source, admission ward, underlying comorbidities, and previous invasive procedures within 30 days of admission; the 7-day, 30-day, and all-causes mortality rates were examined. Furthermore, the candidemia cases were retrieved from all *Candida* infections, and their demographic and clinical characteristics were analyzed separately.

### Definitions.

Patients aged 1 to 28 days are neonatal, >28 days to 1 year are infants, 2 years to 16 years are children, 17 years to 65 years are adults, and greater than 65 years are older patients. Candidemia was defined as at least one positive blood culture of *Candida* in the blood samples collected from peripheral or central bloodlines, along with signs and symptoms of *Candida* infection. Data analysis was limited to the first positive culture for patients who had multiple positive cultures for *Candida* species. Data on previous invasive procedures were recorded for events that occurred within 30 days prior to the onset of *Candida* infection.

### *Candida* isolation, identification, and susceptibility testing.

The *Candida* species were isolated from the biological specimens of patients who visited the hospital, and the following standard isolation protocols were observed: direct microscopy with potassium hydroxide, aerobic and anaerobic incubation in BacT/AlerT 3D vials (Bruker Diagnostics, Inc., USA) (for blood samples), and culturing on CHROMagar-*Candida* medium (63). Matrix-assisted laser desorption ionization–time of flight mass spectrometry (MALDI-TOF MS) was used for the species identification of *Candida* using the MALDI Biotyper RTC 4.0 package (Bruker Daltonik). Antifungal susceptibility testing for five drugs (amphotericin B, 5-flucytosine, fluconazole, itraconazole, and voriconazole) was performed using the ATB FUNGUS 3 kit (bioMérieux, France) following the manufacturer’s guidelines. C. krusei ATCC 6258 and C. parapsilosis ATCC 22019 were used as the quality control strains. The results of susceptibility testing were interpreted according to Clinical & Laboratory Standards Institute (CLSI) guidelines (64, 65).

### Statistical analysis.

The data from the hospital’s electronic medical records were collected by two researchers (D.Z.) and (C.C.) independently using Excel sheets (2021). The two files were subsequently compared to identify and avoid any possible errors. The categorical variables were reported as absolute numbers and relative percentages, while the quantitative variables were presented as median and interquartile ranges. The univariate analysis of baseline characteristics for infection with C. albicans and NCA was performed by Chi-square test. To analyze the risk factor for candidemia, the odd ratios with 95% confidence intervals (CI) and *P* values were determined, considering *Candida* infections other than bloodstream infections (BSI) as the control. The 95% confidence intervals for odd ratios were calculated using the Baptist-Pike method, while the *P* values were derived using the chi-square test. *P* values of less than 0.05 were considered statistically significant and were determined by two-tailed tests. All the statistical analyses and graphical visualizations were performed using GraphPad Prism v.8.0.2.

### Ethics approval.

Ethics approval was provided by the Human Research Ethics Committee of the hospital (reference no. 2021-C-106) following the Declaration of Helsinki criteria. Consent forms from the patients were waived by the ethics committee, as all the clinical samples were obtained from the hospital laboratory as routine work and not for this study.

### Data availability.

All the data are presented in the manuscript; raw data are available by request to the first author (bilal.microbiologist@yahoo.com).
